# Ocular fundus manifestation of two patients following long-term chloroquine therapy: a case report

**DOI:** 10.1186/1746-1596-5-20

**Published:** 2010-03-29

**Authors:** Xiaoyun Ma, Liang Yan, Linping He, Dongyi He, Hao Lu

**Affiliations:** 1 Department of Ophthalmology, Guanghua Rheumatoid Arthritis Specialized Hospital, Shanghai, China; 2 Department of Ophthalmology, Shanghai Baoshan Central Hospital, Shanghai, China; 3 Department of Internal medicine, Guanghua Rheumatoid Arthritis Specialized Hospital, Shanghai, China

## Abstract

This report describes the typical manifestations of chloroquine retinopathy with some advanced new technology. A series of examinations were performed on the patients, including the fundus fluorescein angiography, optical coherence tomography, GDxVCC Nerve Fiber Analyzer, full-field electroretinography, multifocal electroretinography and visual field examinations, to provide a better understanding of chloroquine retinopathy.

## Introduction

Chloroquine has long been used in the treatment and prevention of malaria[1-4], and for a long time, the agent is still being used to treat other diseases. For example, due to its immunosuppressive effects, chloroquine has been used in the treatment of autoimmune disorders, such as rheumatoid arthritis and lupus erythematosus. Chloroquine has also been tested in clinical trials as an antiretroviral agent against HIV [5] and as a radiosensitizer and chemosensitizer for human cancer therapy [6]. However, the side effects of Chloroquine, including ocular toxicities, limit its long-term use [2-4,6].

Herein we describe two patients who developed severe ocular toxicity following long-term chloroquine treatment. The clinical characteristics of their retinopathy were extensively investigated using various techniques, and are reported in this manuscript. We are not trying to provide information which would help in the early screening for chloroquine retinopathy, but to describe the typical manifestations of chloroquine retinopathy with some advanced new technology which have not been applied on this disease before, believing that it would provide a better understanding of chloroquine retinopathy.

## Case Reports

### Case 1

**Figure 1:**
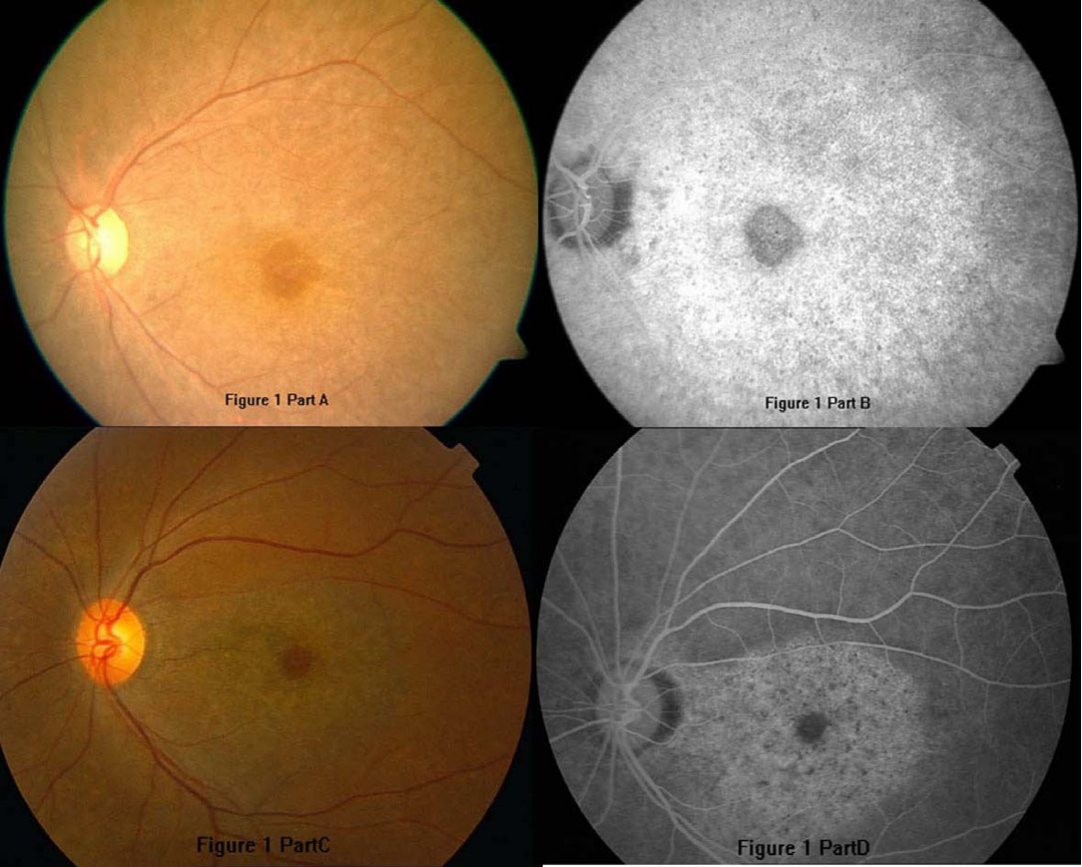
**The ocular fundus photographs and fundus fluorescein angiography (FFA) of the two patients**. A and B: the left eye of case 1; C and D: the left eye of case 2. There were pigmentary deposits and atropy in the whole retina. The hyperfluorescence in FFA images enhanced by time, without leakage.

**Figure 2:**
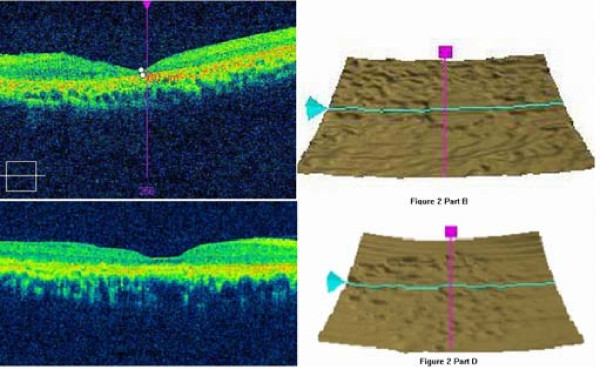
**The optical coherence tomography (OCT) scan of the two patients**. A: severe macular thinning in Case 1. B: Irregular retinal pigment epithelium (RPE) signal in Case 1. C: neurepithelium atropy in Case 2. D: Irregular RPE signal in Case 2.

**Figure 3:**
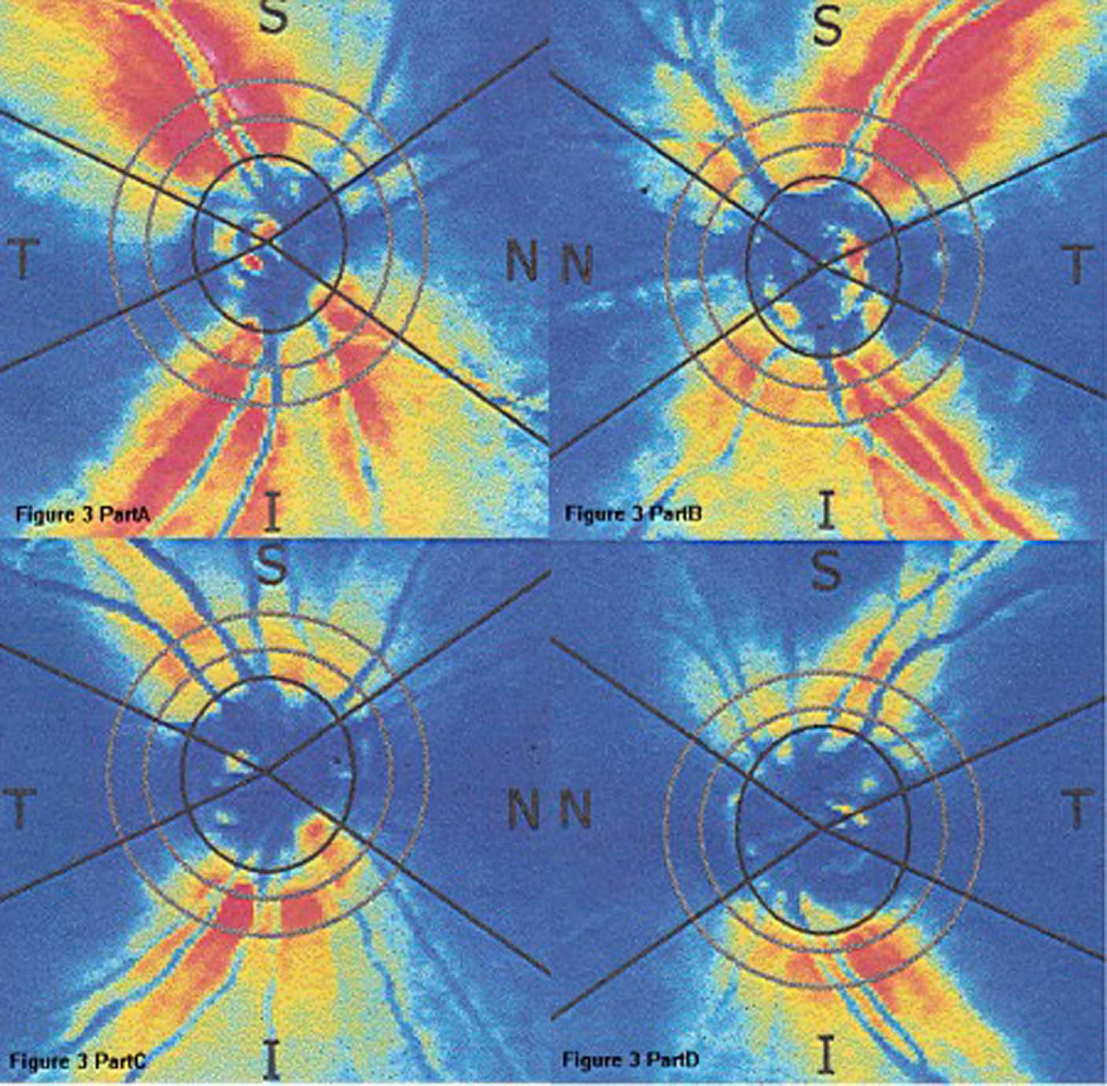
**The retinal nerve fiber layer (RNFL) thickness image of GDxVCC around the optic nerve, the thickness values were normal**. A: the right eye of case 1. B: the left eye of case 1. C: the right eye of case 2. D: the left eye of case 2.

**Figure 4:**
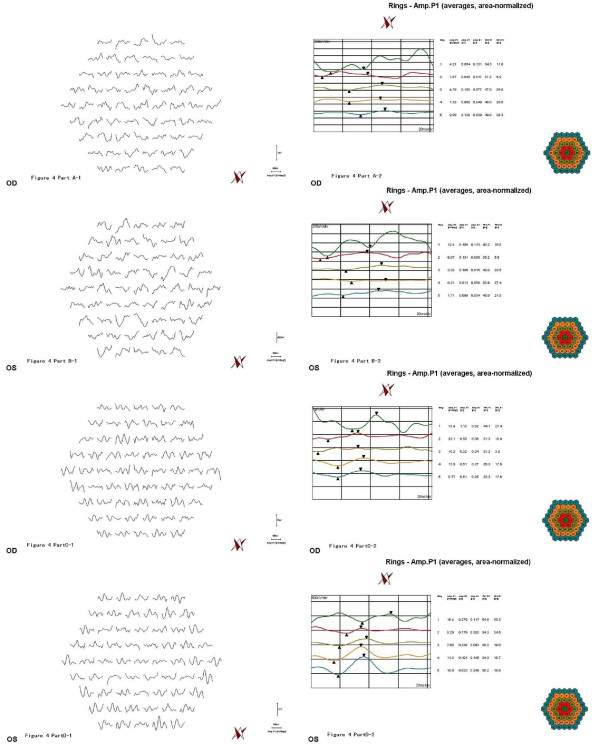
**Multifocal electroretinography (mfERG) of the two patients**. A: the right eye of case 1. B: the left eye of case 1. C: the right eye of case 2. D: the left eye of case 2. The images indicated reduced focal responses with a decrease in amplitude and prolonged peak latency of N1 and P1 responses in both eyes. In case 1, the amplitude of N1 and P1 of ring 1,2,3,4,5 remarkably diminished, and the peak latency of N1 and P1 responses were both delayed. In case 2, there were a decrease in amplitude and prolonged peak latency of N1 and P1 responses in both eyes, especially in the ring 1 and 2.

A 43-year-old woman had taken chloroquine for her rheumatoid arthritis for 16 years, and had received a total dose of approximately 800 g of the drug (250 mg daily for two years, then 250 mg alternative daily for 14 years). Her visual acuity had decreased gradually during the previous 5 years. At her latest examination, her visual acuity was 20/100 OD and hand motions OS, Goldmann applanation intraocular pressure was normal (OD16 mmHg, OS 16 mmHg), but the center visual field (10-2 Humphrey Visual Field Analyzer, Humphrey Instruments Inc, San Leandro, Calif) was lost, and the Goldmann visual field showed a temporal visual island. Photography of her ocular fundus showed pigmentary deposits and atrophy, attenuation of the retinal artery, waxy pallor of the optic disc, and retinal atrophy (Figure 1 Part A). Fundus fluorescein angiography (FFA) showed a marked window defect in the macular area, blocked fluorescence in the central fovea, and dispersed blocked fluorescence dots in the peripheral retina (Figure 1 Part B), but the shape and the boundary of the lesion in the macula area were not so clear, compared to the pictures shown in the textbook or other reports. The optical coherence tomography (OCT, Cirrus HD OCT, SW Ver:3.0.0.64, Carl Zeiss Meditec, Inc. Germany) indicated obvious thinning of the macula and the proliferation of the retinal pigment epithelium (RPE) in the central fovea with adjacent RPE atrophy (Figure 2 Part A). In addition, the RPE signal was irregular (Figure 2 Part B). The retinal nerve fiber layer (RNFL) thickness around the optic nerve measured by OCT was normal (figure not shown). Additionally, examination using a GDxVCC Nerve Fiber Analyzer (Carl Zeiss Meditec, Dublin, CA, USA) showed no abnormality of the RNFL thickness around the optic nerve (Figure 3 Part A and B). The full-field electroretinography (FERG) amplitudes were non-recordable (recorded according to the international standard [7]). The multifocal electroretinography (mfERG) were recorded binocularly with a stimulus size of 61 hexagons using the RETIscan System (Roland Consult, Wiesbaden, Germany) in accordance with the guidelines of the International Society for Clinical electrophysiology of Vision (ISCEV) [8]. The data of amplitude and peak latency of N1 and P1 response from ring 1 to 5 of both eyes were shown in Figure 4. The results demonstrated reduced focal responses with a decrease in amplitude (Figure 4 Part A and B), the amplitude of N1 and P1 of ring 1,2,3,4,5 remarkably diminished, and the peak latency of N1 and P1 responses were both delayed.

### Case 2

A 55-year-old woman had taken chloroquine for rheumatoid arthritis for more than 4 years, with an estimated total dose of 365 g (250 mg daily). She first presented with a history of gradually decreasing visual acuity without any other symptoms approximately two years earlier. The latest examination revealed that her visual acuity was 30/200 OD and 60/200 OS, Goldmann applanation intraocular pressure was normal (OD13 mmHg, OS12 mmHg), the center visual field was severely damaged, and the Goldmann visual field showed central scotoma in both eyes. Her ocular fundus photograph also showed pigmentary deposits and atrophy, attenuation of the retinal artery, waxy pallor of the optic disc, and retinal atrophy (Figure 1 Part C). Differing from Patient 1, FFA of Patient 2 showed marked hyperfluorescence in the macular area with clear demarcations (Figure 1 Part D). Round-shaped blocked fluorescence was seen in the central fovea. OCT showed retinal nerve fiber layer atrophy and irregular RPE signals (Figure 2 Part C and D), however, the central fovea thickness was not decreased to as great an extent as that observed in Patient 1. The RNFL thickness around the optic nerve measured by OCT was normal, which was also supported by the GDxVCC results (Figure 3 Part C and D). The FERG amplitudes were decreased in both eyes, and the mfERG (with 61 stimuli) indicated reduced focal responses with a decrease in amplitude and prolonged peak latency of N1 and P1 responses in both eyes, especially in the ring 1 and 2 (Figure 4 Part C and D). The data of amplitude and peak latency of N1 and P1 response from ring 1 to 5 were shown in Figure 4 Part C and D.

## Discussion

Chloroquine toxicity is not uncommon, especially in patients receiving long-term therapy [1-3]. While hydroxychloroquine is now frequently prescribed instead of chloroquine because of its higher therapeutic window, its use has also been shown to cause retinal damage [4]. Histological analysis of human and animal retinas with chloroquine toxicity has demonstrated the loss of ganglion cells, photoreceptors, and RPE, which likely result from inhibition of lysosomal phospholipases and protein synthesis. However, the initial retinal damage is thought to occur in ganglion cells [7].

In contrast to the usual clinical observations, there was no major pigment atrophy ring with a clarified boundary around the central fovea (making the characteristic bulls-eye) in the FFA image from our patients. However, we did observe a marked hyperfluorescence in the macular area, possibly due to RPE atrophy. Although it is frequently used in the clinic, full-field ERG may not be a reliable method for identifying early chloroquine retinopathy [3]. In our cases, because the two patients had severe retinopathy, the FERG amplitudes were non-recordable. Therefore, F-ERG analysis may have limited value in the diagnosis of early chloroquine retinopathy, because the toxicity is localized and a full field analysis may not be sensitive to localized retinal toxocity in early disease.

It has been suggested that mfERG is a more sensitive method that can be used to detect chloroquine retinopathy [9]. For example, in one study, even though clinical examinations found no abnormalities in a patient with a long history of hydroxychloroquine use [3], mfERG showed an absence of foveal rings and the presence of pericentral isoelectric waveforms. In our current report, the patients had severely damaged visual acuity and center visual field loss, mfERG showed a reduction in focal responses and a delay in the peak latency.

There have been some reports in which OCT was used in the detection of chloroquine and hydroxychloroquine-induced retinopathy [10]. In our cases, not only the "bulls-eye" was shown in the fundus photograph, but also the proliferation-atrophy-proliferation changes of the RPE in the macular area were detected by OCT. Confirming these results, the RNFL thickness measured by GDx (GDx Nerve Fiber Analyzer (NFA) is a relatively new and improved technology that uses scanning laser polarimetry (SLP)) was within the normal range in our patients, this result was different from the conclusions of some other reports. For example, Bonanomi et al [11] reported that mean RNFL thickness measured by GDxFCC from patients using chloroquine were significantly different from the control group in all regions around the optic disc. Possible explanation for this difference might be that the damage of retina in some patients taking chloroquine distributed mainly in the macula area, while the RNFL thickness around the optic disc remained normal.

To the best of our knowledge, this is the first report describing the findings of FFA, OCT, GDxVCC NFA, FERG, mfERG and visual field examinations in the same patients with chloroquine retinopathy. While the fundus pictures from the two patients are similar, there are differences in their macular thickness. Thus, given the differences found using the various methods and instruments, and the well-documented weaknesses of the various techniques, it is evident that multiple analyses are helpful for better understanding of chloroquine retinopathy.

## Consent

Written informed consent was obtained for publication of these two case reports and accompanying images. Copies of the written consent are available for review by the Editor-in-Chief of this journal.

## Competing interests

The authors declare that they have no competing interests.

## Authors' contributions

XM, LY and LH performed ophthalmic examinations, conducted the design of the study and drafted the manuscript. DH participated in the design of the study and drafted the manuscript. HL participated in the electrophysiology examinations and drafted the manuscript. All authors read and approved the final manuscript.
